# Wavelet-Based Spectral Feature Gating for Near-Infrared Remote Photoplethysmography Estimation

**DOI:** 10.3390/s26185734

**Published:** 2026-09-09

**Authors:** Gyutae Hwang, Sang Jun Lee

**Affiliations:** Division of Electronic and Information Engineering, Jeonbuk National University, Jeonju 54896, Republic of Korea; gyutae741@jbnu.ac.kr

**Keywords:** deep learning, computer vision, physiological measurement, remote photoplethysmography

## Abstract

Near-infrared (NIR) video-based remote photoplethysmography (rPPG) enables physiological measurement under low-light conditions using monochrome facial videos. NIR videos lack chrominance-based cues, while weak cardiac variations remain entangled with motion, illumination, and imaging noise. To address these limitations, we propose a wavelet-based spectral feature gating (WSFG) module for NIR-based rPPG estimation. The WSFG module applies a two-level stationary wavelet transform (SWT) to decompose intermediate temporal features without temporal downsampling. Specifically, learnable spectral gates regulate each sub-band contribution and reconstruct branch-specific temporal features through inverse SWT. Three specialized branches preserve cardiac variations, suppress nuisance components, and model relative optical attenuation from low-frequency reference features. Experiments on egoPPG-DB dataset demonstrate that the proposed method achieves the lowest mean absolute error (MAE) and mean absolute percentage error (MAPE) of 8.56 bpm and 9.86%, respectively. Further analysis highlight the effectiveness of frequency-aware feature modeling for NIR-only rPPG estimation.

## 1. Introduction

Remote photoplethysmography (rPPG) estimates heart rate by capturing subtle blood-volume changes in the skin using a camera [1]. Heart rate and heart rate variability reflect physiological changes associated with fatigue and drowsiness and can support early detection of hazardous states during driving [2]. Conventional rPPG studies have mainly estimated pulse signals from RGB facial videos by analyzing color variations induced by blood-volume changes [3]. Recent deep learning methods learn spatiotemporal representations to improve robustness against motion and illumination changes, substantially advancing rPPG estimation performance [4,5]. Recent multimodal physiological sensing studies have also combined optical imaging with complementary cardiorespiratory and thermal measurements to improve the robustness of physiological and affective state monitoring [6,7]. However, RGB-based rPPG signals remain sensitive to ambient illumination, making stable measurement difficult under low-light or rapidly changing lighting conditions [8]. Near-infrared (NIR) imaging enables physiological measurement beyond RGB conditions, while NIR eye-tracking (ET) cameras in egocentric systems continuously observe the periocular region [9,10]. Accordingly, existing ET videos can support continuous heart rate estimation in egocentric settings without requiring additional physiological sensors [11]. Although NIR-based rPPG offers practical advantages for low-light and egocentric monitoring, its measurement accuracy remains constrained by weak pulsatile variations and motion sensitivity. Therefore, further improvements in sensing robustness and estimation accuracy are required for practical physiological monitoring.

Despite these advantages, pulsatile variations observed in NIR videos are relatively weak and may exhibit low signal-to-noise ratio (SNR), making stable rPPG estimation challenging [12]. Moreover, monochrome NIR imaging provides limited chromatic information for separating cardiac variations from motion- and illumination-induced intensity changes [13]. Cardiac pulsations exhibit quasi-periodic structures within specific frequency ranges, whereas interfering variations can occupy different spectral regions and temporal patterns [14]. Directly processing these mixed temporal features may therefore propagate dominant non-cardiac variations into the learned cardiac representation. Frequency decomposition separates the mixed representation into spectral sub-bands, enabling cardiac and interfering components to be examined at different frequency ranges. Wavelet transforms further preserve temporal localization during spectral decomposition, allowing transient variations and periodic cardiac patterns to remain distinguishable [15]. Therefore, frequency-aware feature decomposition can be considered for NIR-based rPPG estimation to separate cardiac and nuisance components before selectively integrating informative spectral features [12,14,16].

Recent rPPG studies have incorporated frequency information through frequency-domain supervision and wavelet-based feature decomposition to exploit the quasi-periodic structure of physiological signals. Supervised methods [5,17,18] align predicted Power spectral density (PSD) or frequency distributions with references, whereas unsupervised methods [16,19] encourage physiological peaks and spectral sparsity. However, these objectives constrain output spectra without explicitly decomposing or selecting spectral components mixed within intermediate features. For NIR videos, weak pulsatile variations and low SNR may limit output-level spectral supervision for isolating cardiac features. Meanwhile, discrete wavelet transform (DWT) has been embedded within rPPG models to decompose intermediate features into low- and high-frequency components [20]. However, DWT downsamples filtered coefficients, causing temporal resolution to decrease progressively across deeper decomposition levels. Thus, feature-level frequency analysis preserving temporal resolution while selectively weighting cardiac and nuisance sub-bands can be considered for NIR-based rPPG estimation.

To address these limitations, stationary wavelet transform (SWT) [21] decomposes signals into multiple frequency bands without temporal downsampling. This property is particularly relevant to rPPG estimation methods, where camera frame rates provide relatively limited temporal sampling for subtle pulsatile variations [22]. SWT recursively convolves features with level-dependent low-pass and high-pass filters expanded by inserting zeros between filter coefficients. This à trous filtering removes the downsampling operation, allowing every approximation and detail coefficient sequence to preserve the input temporal dimension across all levels. Based on this property, we propose a wavelet-based spectral feature gating (WSFG) module, which decomposes temporal NIR features into multiple spectral sub-bands. Specifically, the cardiac branch emphasizes spectral components associated with cardiac pulsation, whereas the nuisance branch suppresses components interfering with rPPG estimation. Each branch uses learnable spectral gates to adaptively weight decomposed components instead of relying on fixed frequency-band selection. Through branch-specific gating, the WSFG module preserves cardiac information while controlling nuisance contributions without further reducing temporal resolution.

Given the limited physiological cues observable in NIR videos, we design an additional WSFG branch to model attenuation-related low-frequency intensity variations. Photoplethysmography (PPG) signals consist of pulsatile AC components and slowly varying DC components, with the latter containing low-frequency intensity variations [23]. In biological tissue, light is attenuated by absorption and scattering, while the modified Beer–Lambert law [24] relates optical attenuation changes to absorption changes under stable scattering. The low-frequency component can capture the slowly varying intensity baseline on which pulsatile AC variations are overlaid. A logarithmic transformation converts multiplicative attenuation into additive variations, making relative changes in optical attenuation easier to model. Accordingly, the Attenuation branch applies log-domain modeling to low-frequency SWT components rather than estimating absolute absorption coefficients. These attenuation-related features complement the Cardiac branch by providing the optical context in which weak pulsatile variations are observed.

The main contributions of this work are summarized as follows:

- We propose a Wavelet-Based Spectral Feature Gating (WSFG) module that decomposes NIR temporal features into spectral sub-bands while preserving temporal resolution.
- We design specialized Cardiac and Nuisance branches with learnable spectral gates to selectively emphasize cardiac components and suppress interfering frequency components.
- We introduce an Attenuation branch that applies log-domain modeling to low-frequency SWT components to learn attenuation-related characteristics complementary to cardiac features.

The remainder of this paper is organized as follows: Section 2 reviews related work. Section 3 presents the proposed methodology. Section 4 reports the experimental results. Finally, Section 5 concludes the paper.

## 2. Related Work

### 2.1. RGB-Based Remote Photoplethysmography

RGB-based rPPG estimation methods have evolved through diverse spatiotemporal architectures to capture subtle color variations and temporal dynamics from facial videos. Early deep learning approaches [4,25,26] employed appearance-guided attention, temporal shifts, and 3D spatiotemporal convolutions to model local skin variations and temporal dynamics. Transformer-based methods [5,17] subsequently utilized temporal differences and periodic attention to capture long-range dependencies and quasi-periodic patterns in rPPG signals. Recent approaches further incorporate state-space modeling and multidimensional feature factorization for efficient temporal modeling and physiological feature extraction [27,28,29]. However, these methods are primarily designed for RGB videos, where chrominance variations provide strong physiological cues. In contrast, monochrome NIR videos lack inter-channel chromatic information and exhibit weaker pulsatile variations, limiting direct adaptation of RGB-oriented feature extraction methods.

### 2.2. NIR-Based Remote Photoplethysmography

NIR-based rPPG estimation methods have utilized structural and optical characteristics and auxiliary motion information to recover weak pulsatile components from single-channel videos. SparsePPG [10] assumes spatially low-rank and frequency-sparse rPPG signals across facial regions, suppressing motion and noise through robust principal component analysis and sparse spectral estimation. Wang et al. [30] exploited distinct discriminative signatures of blood-volume variations and disturbances in NIR videos to separate pulsatile components through least-squares optimization. Hino et al. [31] separated hemoglobin and shading components from two-band NIR videos and applied Wiener estimation to extract pulse waves robust to imaging noise. More recently, PulseFormer [11] estimates cardiac activity from NIR ET videos and introduces the inertial measurement unit (IMU)-based motion-informed temporal attention module using motion cues from egocentric smart glasses. In contrast, our method models learnable low-rank characteristics and explicitly learns cardiac and nuisance frequency components through SWT-based spectral decomposition without external motion information.

### 2.3. Frequency-Domain Analysis for rPPG Signal Estimation

Frequency-domain approaches have integrated spectral transforms into loss functions, input representations, and intermediate feature decomposition to exploit physiological periodicity. Speth et al. [16] constrain spectral peaks and distributions within physiological frequency bands to incorporate cardiac periodicity into model training. Hsu et al. [32] apply the short-time Fourier transform (STFT) to temporal facial signals, generating two-dimensional spectrograms as convolutional neural network (CNN) inputs for rPPG estimation. Zhao et al. [20] embed DWT within neural networks to decompose intermediate features into low- and high-frequency components and learn their temporal–spectral characteristics separately. However, STFT-based representations depend on a fixed window for time-frequency resolution, while DWT progressively reduces temporal resolution through repeated downsampling. Such temporal resolution reduction can hinder the representation of physiological variations in videos with limited frame rates. Therefore, we adopt an SWT-based spectral feature decomposition approach that preserves temporal resolution across all decomposition levels.

## 3. Method

### 3.1. Stationary Wavelet Transform

SWT decomposes temporal features into multiple frequency bands while preserving the original temporal resolution without downsampling. Unlike the DWT, SWT does not decimate coefficients after filtering and instead progressively dilates the low-pass filters (LPFS) and high-pass filters (HPFS) across decomposition levels. Let $x\in R^{T}$ denote a temporal feature of length *T*, with the initial approximation coefficient defined as $A_{0}=x$. We employ the Haar wavelet, where the LPF h and HPF g are defined as

(1) $$h=\frac{1}{\sqrt{2}}\left[1,1\right],\;g=\frac{1}{\sqrt{2}}\left[-1,1\right].$$

The LPF extracts low-frequency approximation coefficients, whereas the HPF extracts high-frequency detail coefficients. At the *j*-th decomposition level, the Haar filters are expanded as $h^{\left(j\right)}=U_{j-1}\left(h\right)$ and $g^{\left(j\right)}=U_{j-1}\left(g\right)$, where $U_{j-1}\left(\cdot \right)$ denotes filter dilation that inserts $2^{j-1}-1$ zeros between adjacent filter coefficients. The dilated LPF and HPF are then convolved with the approximation coefficient from the previous level to obtain the new approximation and detail coefficients,

(2) $$A_{j}=A_{j-1}*h^{\left(j\right)},\;D_{j}=A_{j-1}*g^{\left(j\right)},$$

where ∗ denotes temporal convolution. Since SWT does not perform coefficient downsampling, the temporal dimension remains *T* at every decomposition level: $|A_{j}\left|=\right|D_{j}|=T$. Moreover, SWT consists of fixed convolutional filtering and linear operations, allowing gradients to propagate through the decomposition during end-to-end training. In this work, a two-level SWT decomposes each temporal feature into one approximation band and two detail bands:

(3) $$\mathrm{SWT}\left(x\right)=\left\{A_{2},D_{2},D_{1}\right\}.$$

Here, $A_{2}$ represents the low-frequency band, while $D_{2}$ and $D_{1}$ represent progressively higher-frequency detail-band features. Their identical temporal resolution allows the WSFG module to adaptively weight each spectral band without additional temporal resampling.

### 3.2. Overall Architecture

**Figure 1:**
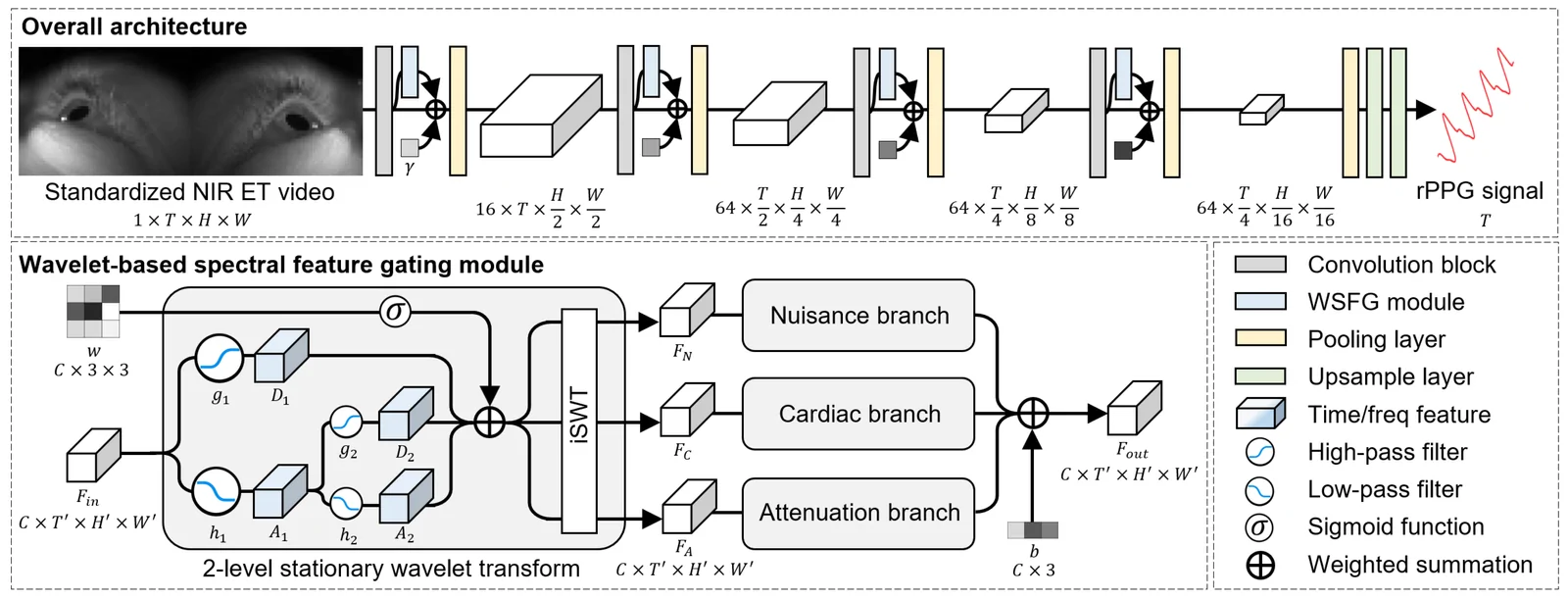
Overview of the proposed rPPG estimation method.

We construct a deep learning framework that combines spatiotemporal feature learning with spectral feature refinement for rPPG estimation from NIR ET videos. As illustrated in Figure 1, the overall architecture consists of a three-dimensional CNN backbone with WSFG modules inserted after each convolution block. The proposed model takes standardized NIR ET video as input and progressively extracts spatiotemporal features through three-dimensional convolutions. Each convolutional feature is processed by a WSFG module to learn cardiac-, nuisance-, and attenuation-related spectral information, which is added through a residual connection. The residual contribution is controlled by a learnable scale γ, initialized with small positive values to limit excessive spectral refinement early in training and gradually increased across deeper layers. Pooling layers progressively reduce the spatial and temporal dimensions, while subsequent upsampling layers restore the temporal resolution. Finally, the reconstructed features are projected to estimate an rPPG signal with the same temporal length as the input video.

### 3.3. Wavelet-Based Spectral Feature Gating

The WSFG module learns the contributions of SWT-decomposed spectral bands to deliver distinct spectral features to the Cardiac, Nuisance, and Attenuation branches. Given an input feature $F_{in}\in R^{C\times T^{\prime }\times H^{\prime }\times W^{\prime }}$, a two-level SWT is applied along the temporal axis to obtain three spectral bands. To selectively assign these bands to each branch, we introduce channel-wise learnable gating weights $w\in R^{C\times 3\times 3}$, where the second and third dimensions correspond to spectral bands and branches, respectively. A sigmoid function is applied to the gating weights to independently control each band contribution between 0 and 1. For frequency band features $Z=[A_{2},D_{2},D_{1}]$ and branch k∈{N,C,A}, the gated feature is defined as

(4) $${\tilde{Z}}_{k}=\sigma \left(w_{k}\right)⊙Z,$$

where $w_{k}\in R^{C\times 3}$ denotes the learnable gating weights for branch *k*, assigning a channel-wise weight to each SWT band, and ⊙ represents element-wise multiplication. The three gated bands assigned to each branch are reconstructed through inverse SWT (iSWT) to generate a branch-specific temporal feature:

(5) $$F_{k}=\mathrm{iSWT}\left({\tilde{A}}_{2,k},{\tilde{D}}_{2,k},{\tilde{D}}_{1,k}\right).$$

The reconstructed feature $F_{k}$ encodes branch-specific spectral characteristics determined by its respective gating weights. These features are subsequently processed by the Nuisance, Cardiac, and Attenuation branches. The gating weights w are initialized with branch-specific spectral priors, according to the objectives of the Nuisance, Cardiac, and Attenuation branches, while retaining flexibility to adapt during training.

**Figure 2:**
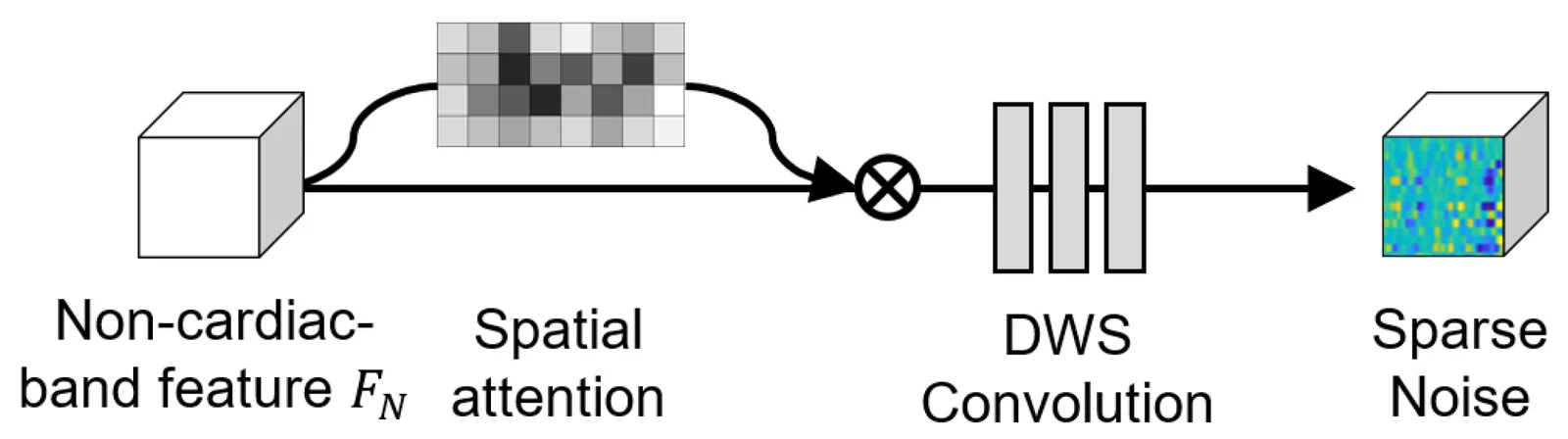
Architecture of the nuisance branch.

**Figure 3:**
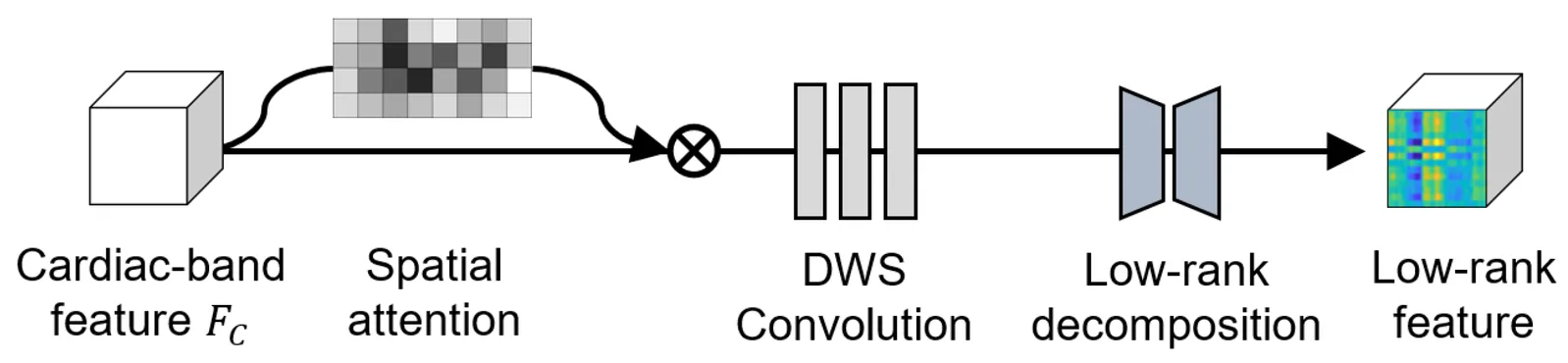
Architecture of the cardiac branch.

**Figure 4:**
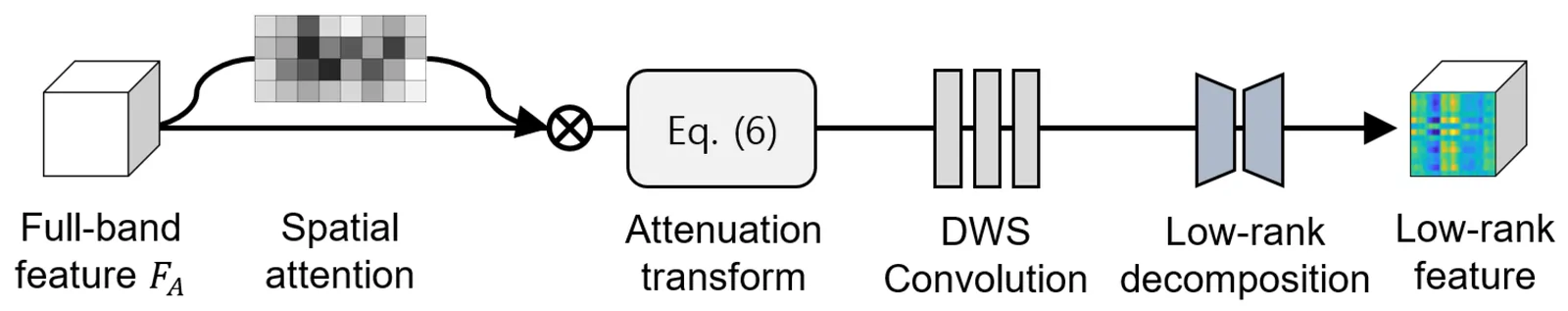
Architecture of the attenuation branch. Eq. denotes the corresponding equation in the text.

The reconstructed branch-specific features output distinct spectral and spatial characteristics according to the objective of each branch. Given the temporal sampling frequency $f_{s}$, the two-level SWT bands $A_{2}$, $D_{2}$, and $D_{1}$ approximately correspond to $\left[0,f_{s}/8\right]$, $\left[f_{s}/8,f_{s}/4\right]$, and $\left[f_{s}/4,f_{s}/2\right]$, respectively. As illustrated in Figure 2, Figure 3 and Figure 4, all branches share a common processing structure consisting of spatial attention and depthwise separable (DWS) convolution. Branch-specific spatial attention enables each branch to independently learn spatial characteristics relevant to its designated objective. DWS convolution efficiently refines features using sequential temporal and spatial depthwise convolutions followed by pointwise channel integration. SparsePPG [10] separates physiological and disturbance components by exploiting low-rank cardiac structures shared across skin regions and sparse local disturbances. Inspired by this perspective, the proposed WSFG module learns low-rank and sparse characteristics as branch-specific feature representations rather than explicit decompositions.

#### 3.3.1. Nuisance Branch

As illustrated in Figure 2, the nuisance branch extracts non-cardiac spectral components associated with non-physiological variations. It models temporally and spatially irregular disturbances, including motion, illumination variation, and imaging noise. These disturbances are represented as sparse components to form a learnable sparse noise distinct from cardiac features. The resulting nuisance feature serves as a subtraction term in gated spectral feature fusion to suppress non-physiological variations.

#### 3.3.2. Cardiac Branch

The Cardiac branch extracts physiological variations from spectral components concentrated within the cardiac band, as shown in Figure 3. Cardiac-induced blood flow changes appear coherently across multiple skin regions, motivating a spatial low-rank representation. Accordingly, a learnable low-rank decomposition with fixed rank (r=4) is applied to the refined features. The resulting cardiac feature serves as the primary physiological component for gated spectral feature fusion and rPPG estimation.

#### 3.3.3. Attenuation Branch

In NIR imaging, physiological intensity variations are weak and easily obscured by ambient illumination and surface reflections. To compensate for this limitation, the attenuation branch uses full-band features to learn relative optical attenuation changes, as shown in Figure 4. Let $F_{A}^{\prime }$ denote the feature after spatial attention. We extend the Beer–Lambert relation $A=-\log\left(\frac{I}{I_{0}}\right)$ to the feature domain. We utilize $\mathrm{LPF}(F_{A}^{\prime })$ as a slowly varying temporal reference to measure relative changes from the current feature. The resulting attenuation feature is defined as

(6) $$F_{A}^{att}=-\log\left(\frac{F_{A}^{\prime }}{\mathrm{LPF}\left(F_{A}^{\prime }\right)}\right).$$

This log-domain transformation emphasizes relative attenuation changes against the reference rather than absolute feature intensity. Similar to cardiac features, attenuation changes can form spatially correlated patterns across neighboring skin regions with similar illumination and tissue absorption properties. Therefore, the attenuation branch further models temporal attenuation patterns using a learnable low-rank feature.

#### 3.3.4. Gated Spectral Feature Fusion

The three branch features contain distinct spectral characteristics and are integrated through gated spectral feature fusion. Let ${\tilde{F}}_{N}$, ${\tilde{F}}_{C}$, and ${\tilde{F}}_{A}$ denote the nuisance, cardiac, and attenuation branch features, respectively. Their contributions are independently controlled by learnable branch weights $b_{k}$. Cardiac and attenuation features are combined additively, while the nuisance feature serves as a subtraction term. The fused feature is defined as

(7) $$F_{\mathrm{fuse}}=-b_{N}{\tilde{F}}_{N}+b_{C}{\tilde{F}}_{C}+b_{A}{\tilde{F}}_{A},\;b_{k}=\frac{\exp(\beta_{k}/\tau )}{\sum_{j}\exp\left(\beta_{j}/\tau \right)},$$

where $\beta_{k}$ denotes the learnable branch logit and τ=0.5 is the temperature parameter for Softmax normalization. This fusion learns the relative contribution of each branch to construct features for subsequent rPPG estimation.

### 3.4. Loss Function

We employ the Negative Pearson correlation loss to optimize temporal consistency between predicted rPPG and ground-truth (GT) PPG signals [25]. This loss is designed to maximize the Pearson correlation between the two signals. It therefore emphasizes temporal waveform changes and relative shape consistency rather than absolute signal magnitude. Training minimizes this loss to improve the temporal waveform similarity of the predicted rPPG signal. Equation (8) is defined as follows:

(8) $$L=1-\frac{T\sum_{1}^{T}y_{t}{\hat{y}}_{t}-\left(\sum_{1}^{T}y_{t}\right)\left(\sum_{1}^{T}{\hat{y}}_{t}\right)}{\sqrt{\left[T\sum_{1}^{T}y_{t}^{2}-{\left(\sum_{1}^{T}y_{t}\right)}^{2}\right]\left[T\sum_{1}^{T}{\hat{y}}_{t}^{2}-{\left(\sum_{1}^{T}{\hat{y}}_{t}\right)}^{2}\right]}},\;t\in \left\{1,\ldots ,T\right\}.$$

where y and $\hat{y}$ denote the GT PPG and predicted rPPG signals, respectively.

## 4. Results

### 4.1. Dataset

We use the latest benchmark dataset egoPPG-DB [11] to evaluate NIR-based rPPG estimation. The egoPPG-DB dataset consists of approximately 13 h of ET videos and synchronized physiological signals collected from 25 participants. ET videos were captured using monochrome NIR cameras on Project Aria glasses at a resolution of 320×240 pixels per eye and 30 frames per second. GT PPG was measured at 128 Hz using contact PPG sensors mounted on the nose pads. Electrocardiogram (ECG) and IMU signals were also recorded at 1024 Hz and excluded from both training and inference. Participants performed various daily activities, including video watching, office work, kitchen work, dancing, exercise bike, and walking. These activities range from stable conditions to dynamic scenarios involving substantial motion and heart rate variations, with overall heart rates spanning 44–164 bpm. We evaluate rPPG estimation methods that use only NIR ET videos as their inputs.

### 4.2. Implementation Details

All experiments were conducted on a system equipped with an Intel Core i9-10940X CPU, 64 GB DDR4 RAM, and two NVIDIA GeForce RTX 3090 Ti GPUs. The software environment consisted of Ubuntu 20.04, Python 3.11.9, and PyTorch 2.4.0. The model was trained with a learning rate of 0.0009 and a batch size of 8. Input NIR video clips contained 128 frames with spatial dimensions of 48 × 128 pixels, and standardization was applied. Following the official subject-independent evaluation protocol, predicted rPPG signals were first filtered using a Butterworth band-pass filter (0.5–2.8 Hz) and then subjected to peak detection over 60-second windows, consistent with the PulseFormer evaluation procedure.

The spectral gates in WSFG were initialized to emphasize using branch-specific spectral priors. At the input sampling frequency of 30 Hz, the $A_{2}$, $D_{2}$, and $D_{1}$ bands approximately correspond to 0–3.75 Hz, 3.75–7.5 Hz, and 7.5–15 Hz, respectively. The initial logits for $\left(A_{2},D_{2},D_{1}\right)$ were set to (−1.5,1.0,1.0) for the Nuisance branch, (1.5,−1.0,−1.5) for the Cardiac branch, and (0,0,0) for the Attenuation branch. These correspond to sigmoid gating values of approximately (0.18,0.73,0.73), (0.82,0.27,0.18), and (0.50,0.50,0.50), respectively. These values provide soft spectral priors for non-cardiac, cardiac, and full-band features, respectively, and all channel-wise spectral gates are subsequently optimized during training.

The residual scales of the layer-wise WSFG modules were initialized to (0.12,0.15,0.18,0.20), while the low-rank decomposition rank was fixed at 4 for the cardiac and attenuation branches.

### 4.3. Evaluation Metrics

We evaluate heart rate estimation performance using mean absolute error (MAE), root mean squared error (RMSE), and mean absolute percentage error (MAPE). MAE measures the average absolute difference between estimated and GT heart rate values in beats per minute (bpm). RMSE measures the square root of the mean squared heart rate error and emphasizes larger estimation errors. MAPE measures the average relative heart rate estimation error with respect to GT and is reported as a percentage (%).

### 4.4. Heart Rate Estimation Results

Table 1 compares the heart rate estimation performance of previous methods with the proposed method. Early two-dimensional CNN methods such as DeepPhys and TS-CAN use spatial attention and temporal modeling and record high MAE of 28.26 and 26.32 bpm on egoPPG dataset. In contrast, PhysNet and PhysFormer explicitly model spatiotemporal features and reduce the MAE to 12.09 and 10.71 bpm, respectively. RhythmFormer, RhythmMamba, and PhysMamba employ periodic attention or state-space modeling and achieve MAE between 13.13 and 15.05 bpm. These results indicate that long-term temporal dependency modeling alone provides limited improvement under NIR-only conditions. PulseFormer was specifically designed for egoPPG-DB and provides the strongest baseline with an MAE of 8.82 and RMSE of 12.03. For a fair NIR-only comparison, we use the Pulse-Former result obtained without IMU input. The proposed method achieves an MAE of 8.56 and MAPE of 9.86 and records the lowest errors for both metrics among all compared methods. These improvements demonstrate the effectiveness of the WSFG module through explicit spectral decomposition and modulation for NIR-only rPPG estimation. However, the second-best RMSE of 12.83 suggests that relatively large errors remain in some samples and require further analysis. Moreover, the reported errors reflect comparative performance within NIR-only contactless heart rate estimation rather than clinical diagnostic accuracy.

### 4.5. Performance Under Different Activities

Table 2 compares activity-specific heart rate estimation performance to analyze the relatively high RMSE observed in the overall evaluation. For Video and Office, the proposed method achieves low MAE of 5.39 and 6.18 bpm and RMSE of 6.95 and 8.00 bpm, respectively. In contrast, Kitchen, Dancing, and Walking involve substantial body and head motion during measurements, resulting in higher MAE of 7.06, 7.82, and 7.10 bpm and corresponding RMSE of 8.73, 9.44, and 11.97. Among all conditions, Bike presents the most challenging scenario, recording an MAE of 22.17 bpm, an RMSE of 27.86, and a MAPE of 19.95%. In summary, these activity-wise results demonstrate that more intensive physical motion leads to larger estimation errors, standing out as a primary contributor to the elevated overall RMSE. In particular, the substantially higher error during the Bike task highlights the limitation of NIR-only sensing under motion-intensive conditions and indicates that further improvements in motion robustness are required for practical physiological monitoring.

### 4.6. Analysis of rPPG Signal Morphology

**Figure 5:**
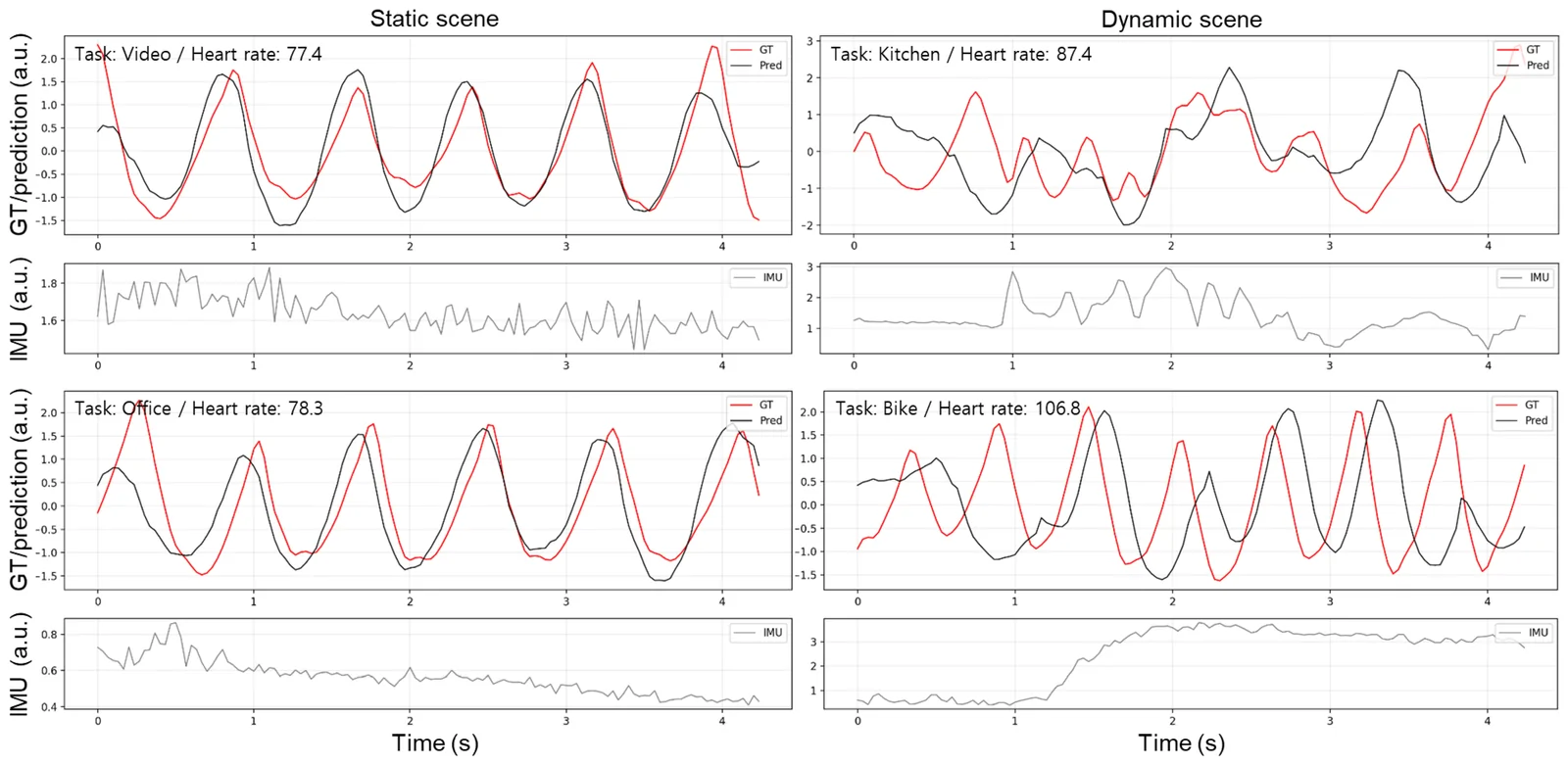
Temporal morphology comparison of standardized GT PPG (red) and predicted rPPG (black) signals. Normalized IMU magnitude (gray) illustrates motion intensity and is not used as model input.

Figure 5 compares temporal waveform morphology of GT PPG and predicted rPPG signals from the same subject across different tasks. In static tasks such as Video and Office, the predicted rPPG signal generally follows the cardiac period and major peaks of PPG signal. Although small amplitude differences and phase delays remain, the periodic waveform morphology associated with cardiac activity is preserved. During the Kitchen task, increased motion introduces noticeable deviations in peak locations and waveform shapes from the PPG signal. In the Bike task, continuous motion produces larger phase and periodicity mismatches with unstable tracking of several cardiac cycles. These waveform deviations coincide with higher IMU magnitudes, indicating that weak NIR cardiac variations are more difficult to estimate during motion-intensive tasks. This observation is consistent with the higher heart rate estimation errors observed for motion-intensive activities in the quantitative results. The accompanying IMU signals are not used as model inputs and only illustrate motion levels across different tasks.

### 4.7. Analysis of Learnable Weights

Table 3 presents the mean and standard deviation across five random seeds for layer-wise branch weights *b* and the learned residual scales γ after training. Softmax-normalized branch weights show distinct distributions across layers, indicating gradual changes in the relative contribution of each branch. In the first layer, the cardiac branch weight is highest at 0.453, supporting early preservation of physiological information in high-resolution features. In the second and third layers, nuisance weights increase to 0.617 and 0.591, emphasizing suppression during deeper feature refinement. The attenuation branch receives lower weights in the early layers and increases to 0.380 in the final layer. This pattern may indicate that attenuation-related information becomes more useful in deeper layers, where slowly varying optical context is represented more stably. The residual scale γ increases from 0.131 and remains larger in deeper layers, allowing WSFG module to contribute progressively after early feature extraction. Notably, the small standard deviations demonstrate the consistency of the layer-wise patterns adaptively learned by the model.

### 4.8. Analysis of Spatial Attention

**Figure 6:**
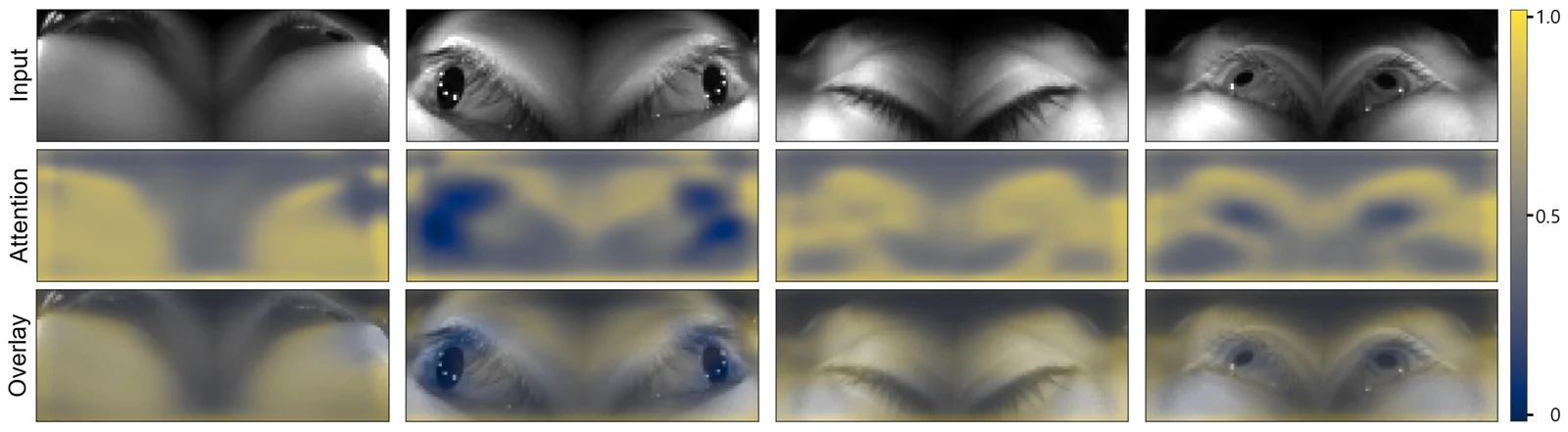
Visualization of spatial attention in the cardiac branch. Blue and yellow indicate lower and higher attention weights, respectively.

Figure 6 presents spatial attention maps and their overlays for four input samples in the cardiac branch. The attention maps assign higher weights to periocular skin regions than to the eyes, eyelashes, and eyelid boundaries. Overexposed regions with strong reflections and excessively dark regions affected by shadows receive lower attention weights. These extreme-intensity regions may not preserve subtle NIR variations associated with cardiac activity. Similar attention patterns are observed across different eye states and gaze directions, indicating consistent emphasis on periocular skin regions. Consequently, the cardiac branch suppresses unstable regions and emphasizes skin features that more reliably retain cardiac-related variations.

### 4.9. Ablation Study

We analyze the effect of different branch combinations on heart rate estimation performance in the proposed method, as shown in Table 4. The individual nuisance, cardiac, and attenuation branches show comparable performance, with MAE of 11.12, 10.99, and 10.94 bpm, respectively. Combining the nuisance branch with the cardiac branch reduces MAE, RMSE, and MAPE by 0.83, 0.39, and 2.18, respectively, compared with the cardiac branch alone. Combining the attenuation branch with the cardiac branch provides larger reductions of 1.76, 1.25, and 3.56 in MAE, RMSE, and MAPE, respectively. Utilizing all three branches achieves the best performance among all configurations. These results indicate that attenuation features complement cardiac features, while nuisance suppression provides additional performance gains in the complete configuration. This finding supports the effectiveness of the branch-specific design based on spectral features for rPPG estimation.

### 4.10. Comparison with Discrete Wavelet Transform

Table 5 compares heart rate estimation performance using DWT and SWT for spectral decomposition using the proposed method. DWT achieves an MAE of 10.50 bpm, RMSE of 14.23, and MAPE of 12.15%. Compared with DWT, SWT reduces MAE by 1.94, RMSE by 1.40, and MAPE by 2.29%. DWT performs downsampling during decomposition, which can reduce temporal resolution and feature alignment. In contrast, SWT preserves the temporal positions of spectral components without downsampling and better retains subtle cardiac variations. These results indicate that SWT is more suitable for spectral feature gating in rPPG estimation.

## 5. Conclusions

In this paper, we proposed a wavelet-based spectral feature gating (WSFG) module to estimate rPPG signals from NIR ET videos. The proposed WSFG module decomposes intermediate temporal features into multiple frequency bands using a two-level SWT. Specifically, learnable spectral gates adaptively regulate the contribution of each decomposed band to the cardiac, nuisance, and attenuation branches. The cardiac branch models spatially coherent physiological variations across skin regions as low-rank features, whereas the nuisance branch extracts non-physiological components such as motion and illumination changes. The attenuation branch learns relative optical attenuation variations using low-frequency reference features and a log-domain transformation. Experimental results demonstrate that the proposed method achieves the lowest MAE and MAPE on the benchmark egoPPG-DB dataset. Branch weight analysis showed a consistent layer-wise progression from cardiac feature preservation to nuisance suppression and attenuation compensation. However, an observed limitation is that strong motion can distort rPPG signal morphology and destabilize phase and periodicity without IMU cues. Future work will improve motion robustness and validate generalization across diverse NIR imaging conditions.

## Figures and Tables

**Table 1:** Heart rate estimation performance of methods evaluated using NIR-only input on the egoPPG-DB dataset. ↓ indicates lower is better; **bold** and underlined values indicate the best and second-best performance, respectively.

Method	MAE ↓	RMSE ↓	MAPE ↓
DeepPhys [4]	28.26	31.97	36.68
TS-CAN [26]	26.32	32.39	29.13
RhythmMamba [28]	15.05	19.78	17.46
PhysMamba [27]	13.94	16.86	17.76
RhythmFormer [17]	13.13	17.43	14.73
PhysNet [25]	12.09	15.43	15.14
PhysFormer [5]	10.71	13.97	12.69
FactorizePhys [29]	10.07	13.43	10.82
PulseFormer [11]	8.82	**12.03**	13.06
Ours	**8.56**	12.83	**9.86**

**Table 2:** Heart rate estimation performance under different activities. ↓ indicates lower is better.

Metric	Video	Office	Kitchen	Dancing	Bike	Walking
MAE ↓	5.39	6.18	7.06	7.82	22.17	7.10
RMSE ↓	6.95	8.00	8.73	9.44	27.86	11.97
MAPE ↓	8.19	8.37	8.67	8.89	19.95	7.60

**Table 3:** Layer-wise branch weights and residual scales learned by the WSFG module. Results are reported as mean ± standard deviation over five random seeds.

Layer	$b_{N}$	$b_{C}$	$b_{A}$	γ
1	0.35±0.06	0.45±0.03	0.19±0.05	0.13±0.02
2	0.62±0.10	0.26±0.07	0.12±0.04	0.27±0.02
3	0.59±0.07	0.17±0.05	0.24±0.07	0.35±0.06
4	0.39±0.06	0.23±0.04	0.38±0.07	0.29±0.03

**Table 4:** Effect of branch combinations. N, C, and A denote the nuisance, cardiac, and attenuation branches, respectively. ↓ indicates lower is better and ✓ indicates that the corresponding component is included during training.

N	C	A	MAE ↓	RMSE ↓	MAPE ↓
✓			11.12	15.26	12.83
	✓		10.99	14.61	13.87
		✓	10.94	14.83	12.33
✓	✓		10.16	14.22	11.69
	✓	✓	9.23	13.36	10.31
✓	✓	✓	8.56	12.83	9.86

**Table 5:** Heart rate estimation performance using DWT and SWT-based spectral decomposition. ↓ indicates lower is better.

Transform	MAE ↓	RMSE ↓	MAPE ↓
DWT	10.50	14.23	12.15
SWT	8.56	12.83	9.86

## Data Availability

Data available in a publicly accessible repository at https://github.com/eth-siplab/egoPPG accessed on 12 August 2026.
